# Inclusion of health equity variables in UK national anaesthesia projects

**DOI:** 10.1111/anae.70031

**Published:** 2025-10-10

**Authors:** Sarah Hudson, Akshay Shah, Søren Kudsk‐Iversen

**Affiliations:** ^1^ Oxford University Hospitals NHS Foundation Trust Oxford UK; ^2^ Imperial College Healthcare NHS Trust London UK

Wider determinants of health have a significant impact on health outcomes [1]. To assist researchers to identify which populations are at higher risk of adverse outcomes, the Cochrane Health Equity Group recommends the use of the PROGRESS‐Plus variables (Place of residence; Race/ethnicity/culture/language; Occupation; Gender/sex; Religion; Education; Socio‐economic status; and Social capital) to identify individual‐level characteristics relevant to equity [2]. This framework, developed through systematic review and expert consensus, has been embedded into equity‐focused reporting guidelines (e.g. PRISMA‐E and CONSORT‐E) and aims to delineate relationships between determinants of health and outcomes. Crucially, including all variables rather than some allows a greater understanding of the intersectional nature of inequity and how having multiple characteristics may compound the disparity.

To complement a broader programme of work assessing the ability of UK peri‐operative health registries to capture PROGRESS‐Plus variables, we sought to assess the degree of inclusion of the wider determinants of health across a range of national observational studies. The National Audit Projects (NAPs) and Sprint National Anaesthesia Projects (SNAPs) are recognised internationally as having had a substantially positive impact on anaesthetic practice and patient outcomes. To complement these, we examined recently completed UK national observational studies with publicly available case record forms: PErioperative CHildhood obesitY (PEACHY); Snapshot Obstetric National Anaesthetic Research project 1 (SONAR‐1); and Patient‐reported Outcomes, Postoperative Pain and pain relief after daY case surgery (POPPY) (online Supporting Information Appendix S1). We reviewed the number of PROGRESS‐Plus variables used in data collection, and where final study reports were available, commented on in the discussion. Ethical approval was not required as the data are publicly available, and no individual patient data were extracted.

The included studies covered a broad range of patient populations, including patients having general surgical procedures, older patients, pregnant women and children. Across NAP and SNAP, we observed a steady increase in PROGRESS‐Plus variables over time, with similar trends across SONAR‐1, POPPY and PEACHY (Table 1). Data on age and sex were included in all, while religion and social capital were included in none. Of the studies, SNAP‐3 and SONAR‐1 were the most inclusive, including socio‐economic status, educational attainment, occupation and residence data in addition to age, frailty, sex and ethnicity.

**Table 1:** PROGRESS‐Plus variables included in NAP, SNAP, PEACHY, SONAR‐1 and POPPY projects.

	Place of residence	Race/ethnicity	Occupation	Gender/sex	Religion	Education	SES	Social capital	Plus
NAP3 (2006)				✓					✓ age
NAP4 (2008)				✓					✓ age
NAP5 (2013)		✓		✓					✓ age
SNAP1 (2014)				✓					✓ age
NAP6 (2016)		✓		✓					✓ age
SNAP2 (2017)		✓		✓					✓ age
PEACHY (2019)				✓		✓			✓ age
NAP7 (2021)		✓		✓					✓ age, frailty
SNAP3 (2022)	✓	✓		✓		✓	✓		✓ age, frailty
POPPY (2025)	✓	✓		✓					✓ age, frailty
SONAR‐1 (2025)	✓	✓	✓	✓		✓	✓		✓ age, disability

PROGRESS‐Plus: Place of residence; Race/ethnicity/culture/language; Occupation; Gender/sex; Religion; Education; Socio‐economic status; and Social capital; NAP, National Audit Project; SNAP, Sprint National Anaesthesia Project; PEACHY, PErioperAtive CHildhood ObesitY; SONAR‐1, Snapshot Obstetric National Anaesthesia Research; POPPY, Patient‐reported Outcomes, Postoperative Pain and pain relief after daY case surgery; SES, socio‐economic status; Plus, personal characteristics and relationships associated with discrimination.

Over time, the number of PROGRESS‐Plus characteristics recorded has increased, and present to a greater degree than what is observed in peri‐operative clinical trials [3]. This may be driven by an increased interest and requirement to explore these associations in our population. As an example of how this trend is important to continue in the upcoming NAP8 and onwards, in multiple studies we have seen disproportionate poor pain management or reduced uptake of regional anaesthesia in patients who are of non‐White ethnic origin; from lower socio‐economic deciles; affected by language barriers; of advancing age; and/or identifying as female [4, 5]. These characteristics are often examined in isolation and in retrospect. In addition, despite census data revealing an impact of employment status and religious affinity on health outcomes [6, 7], these data are collected rarely and their impacts in the peri‐operative setting remain unexplored.

We found that SNAP‐3 and SONAR‐1 showed the feasibility of incorporating over half of the PROGRESS‐Plus variables. This may be in part attributed to patient consent for data collection; however, for studies without consent‐based data, there are still opportunities to collect PROGRESS‐Plus variables through data linkage with hospital episode statistics, the Office for National Statistics and as Indices of Multiple Deprivation through postcodes. Electronic health records can provide many PROGRESS‐Plus variables, although data quality varies and evidence suggests that missing data often affect marginalised groups disproportionately.

An added strength of the NAP studies is the accompanying clinician baseline surveys, which provide a unique opportunity to explore the role of the care provider. Clinicians are themselves community members and political beings and will bring their own bias, either implicit or explicit, which evidence shows is likely to be similar to the wider population [8]. Creating recommendations to address identified equity‐related associations is a complex but crucial task and will require proactive patient and public involvement and engagement which should represent the diverse characteristics of the UK population. Early partnerships between researchers and community members should be created to develop meaningful outcomes and intentionally centring community‐relevant recommendations as a target.

It is reassuring to see that characteristics pertaining to health outcomes are included increasingly in large national observational studies. The current trend must continue, as the broad scope of these projects can provide significant insights into equitable anaesthesia practice. It is vital that future national studies seek wider adoption of PROGRESS‐Plus variables, producing reliable and comprehensive data to assess intersectional inequity in our current healthcare system and provide data for immediate advocacy and to enable prospective work to address growing injustice.

## Supporting information


**Appendix S1.** Included studies.
