# Supplementary material for: Inclusion of health equity variables in UK national anaesthesia projects
Source: Anaesthesia. 2025 Oct 10;81(1):138–9. doi: 10.1111/anae.70031 (PMC12747642; doi:10.1111/anae.70031)
Supplement: Supplementary file 1 — Appendix S1. Included studies. [file ANAE-81-138-s001.pdf]

Appendix S1: Included studies

Abbreviated title (year of data collection)	Main Topic	Link to report or study documents
NAP 3 (2006)	Major Complications of Central Neuraxial Block in the United Kingdom	https://www.rcoa.ac.uk/media/6731
NAP 4 (2008)	Major Complications of Airway Management in the United Kingdom	https://www.rcoa.ac.uk/media/32521
NAP 5 (2013)	Accidental Awareness during General Anaesthesia in the UK and Ireland	https://www.rcoa.ac.uk/nap5-report-publications
NAP 6 (2016)	Anaesthesia, Surgery and Life-Threatening Allergic Reactions	https://www.rcoa.ac.uk/media/33336
NAP 7 (2021)	Perioperative Cardiac Arrest	https://www.rcoa.ac.uk/research/research-projects/national-audit-projects-naps/nap7-report
SNAP 1 (2014)	Patient-reported outcomes after anaesthesia	https://www.rcoa.ac.uk/research/research-projects/sprint-national-anaesthesia-projects-snaps/snap-1
SNAP 2 (2017)	Epidemiology of Critical Care Services	https://www.rcoa.ac.uk/research/research-projects/sprint-national-anaesthesia-projects-snaps/snap-2-epiccs
SNAP 3 (2022)	Frailty and Delirium	https://www.rcoa.ac.uk/snap3-frailty-delirium\

PEACHY (2019)	Prevalence of PErioperAtive CHildhood obesitY in children undergoing general anaesthesia in the UK	https://www.bjanaesthesia.org.uk/article/S0007-0912(21)00548-1/pdf
SONAR-1 (2025)	Snapshot Obstetric National Anaesthesia Research Project 1	https://psrc-cl.nihr.ac.uk/research/snapshot-obstetric-national-approved-documents
POPPY (2025)	Patient-reported outcomes, postoperative pain and pain relief after day case surgery	https://doi.org/10.1111/anae.16460
